# Fatal gunshot injuries in the common buzzard *Buteo buteo* L. 1758 – imaging and ballistic findings

**DOI:** 10.1007/s12024-018-0017-4

**Published:** 2018-08-31

**Authors:** Filip Pankowski, Grzegorz Bogiel, Sławomir Paśko, Filip Rzepiński, Joanna Misiewicz, Alfred Staszak, Joanna Bonecka, Małgorzata Dzierzęcka, Bartłomiej J. Bartyzel

**Affiliations:** 1 Department of Morphological Sciences, Faculty of Veterinary Medicine, Warsaw University of Life Sciences – SGGW, Warsaw, Poland; 2 Toolmark and Ballistic Department, Central Forensic Laboratory of the Police Research Institute, Warsaw, Poland; 3 Virtual Reality Techniques Division, Faculty of Mechatronics, The Institute of Micromechanics and Photonics, Warsaw University of Technology, Warsaw, Poland; 4 University of Zielona Góra, Faculty of Law and Administration, Zielona Góra, Poland; 5 Department of Small Animal Diseases with Clinic, Faculty of Veterinary Medicine, Warsaw University of Life Sciences – SGGW, Warsaw, Poland

**Keywords:** Raptors, Ballistics, PMCT, Bullet, Firearm

## Abstract

The conservation of the common buzzard is assured by the European Union law. In Poland, this wild bird is under strict species protection and it is used as a bioindicator for heavy metals in the environment. A case of the fatal shooting of a buzzard with a firearm by an unidentified shooter is described here. Macroscopic evaluation, X-ray imaging, post-mortem computed tomography, ballistic examination of the isolated bullets and finally a simulation of the assumed position of the bird at the time of the shot were performed. Numerous pellets were found inside the body, together with multiple bone fractures and central nervous system trauma. The buzzard died most probably as a result of spinal cord injury from a single shot that was fired from a smoothbore hunting gun. Collected evidence was insufficient to identify the shooter, which sadly confirms that identification of the perpetrator in wildlife forensics remains low.

## Introduction

Over time, people are increasingly protecting the environment [1]. One of the examples of active protection in Poland is caring for the growth of common buzzard populations as a species that is a bioindicator for heavy metals in the environment [2, 3]. The common buzzard is under strict species protection in Poland. According to the latest data, there are no more than 50 thousand breeding pairs of this bird in the country [4]. Legal regulations in the field of wildlife protection are based both on European Union and national law [5]. There are duly documented cases of animal killing, for example poisoning of 37 dogs and cats in Brazil [6]. Studies describing a diagnostic workup to determine the cause of death of a killed wild animal, especially a bird, are scarce [7]. This article presents a case of the fatal shooting of a buzzard with a firearm. Such events are not uncommon, but rarely result in prosecution. An attempt was made to identify the shooter on the basis of post-mortem examinations of the animal, including diagnostic imaging and ballistic findings.

## Materials and methods

The material was a cadaver of a common buzzard *Buteo buteo* from the Warsaw University of Life Sciences – SGGW, Department of Morphological Sciences museum collection. Firstly, macroscopic examination and palpation were performed. Then, X-ray images were taken in dorsoventral, ventrodorsal and lateral projections using the digital radiography system (Oehm und Rehbein GmbH, 50 kVp, 1.5 mAs). Computed tomography (CT) of the whole animal in the sternal position was performed using a 16-row scanner (Philips & Neusoft Medical Systems NeuViz) with the following parameters: 120 kV, 150 mA, slice thickness 2 mm, reconstructed in a bone algorithm 0.75 mm. Three-dimensional reconstructions of the volumetric image were made in the Vol View 3.4. Bullets were isolated from the body using plastic tools, followed by their photographic documentation with A Canon EOS 70D camera and A Canon EF-S 18-55 mm IS STM lens. A visual inspection of four isolated elements was performed using a stereoscopic microscope (Leica S4E). Measurements were made using a MAUaZ caliper (VIS S.A.) and a weight was obtained using an analytical balance (Radwag AS 110.R2). Based on the data collected, a simulation of the assumed position of the bird at the time of the shot was made. Having the skeleton model obtained from segmentation of radiological data in the open-source program (Blender), the bones were separated and moved so that their position reflected the position taken naturally by the bird and that the bullets in the limbs reflected the flight path of the bullets found in the trunk.

## Findings

### Macroscopic findings

**Fig. 1:**
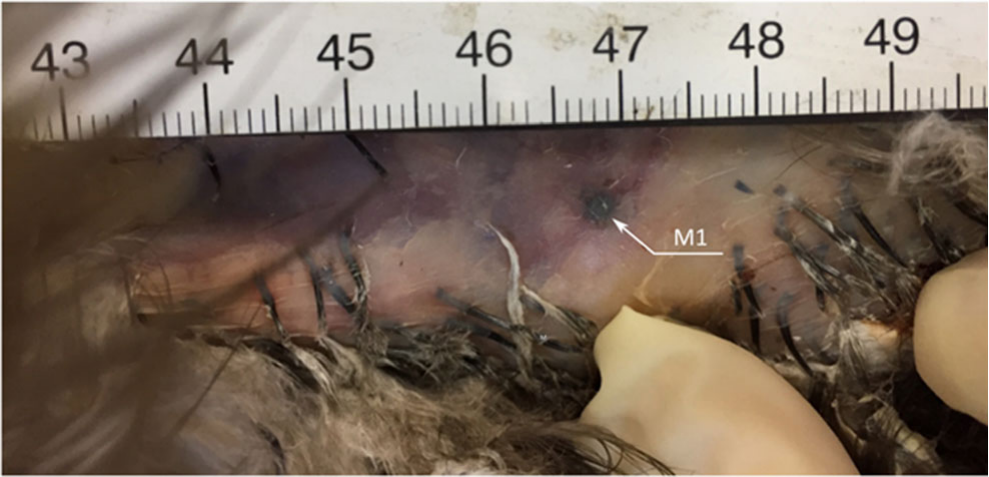
A round metallic element (M1) was seen after removing the feathers on the thorax. It was later isolated from the body for ballistic examination

Blood extravasation, a wound, and a complete fracture were present at the proximal 1/3 of the left radius. In the region of the right penultimate rib, a round, metallic foreign body with a diameter of 4 mm was visible through the skin (Fig. 1). A complete, open, multiple fracture was found at the distal extremity of the left tibiotarsus.

### Radiologic findings

**Fig. 2:**
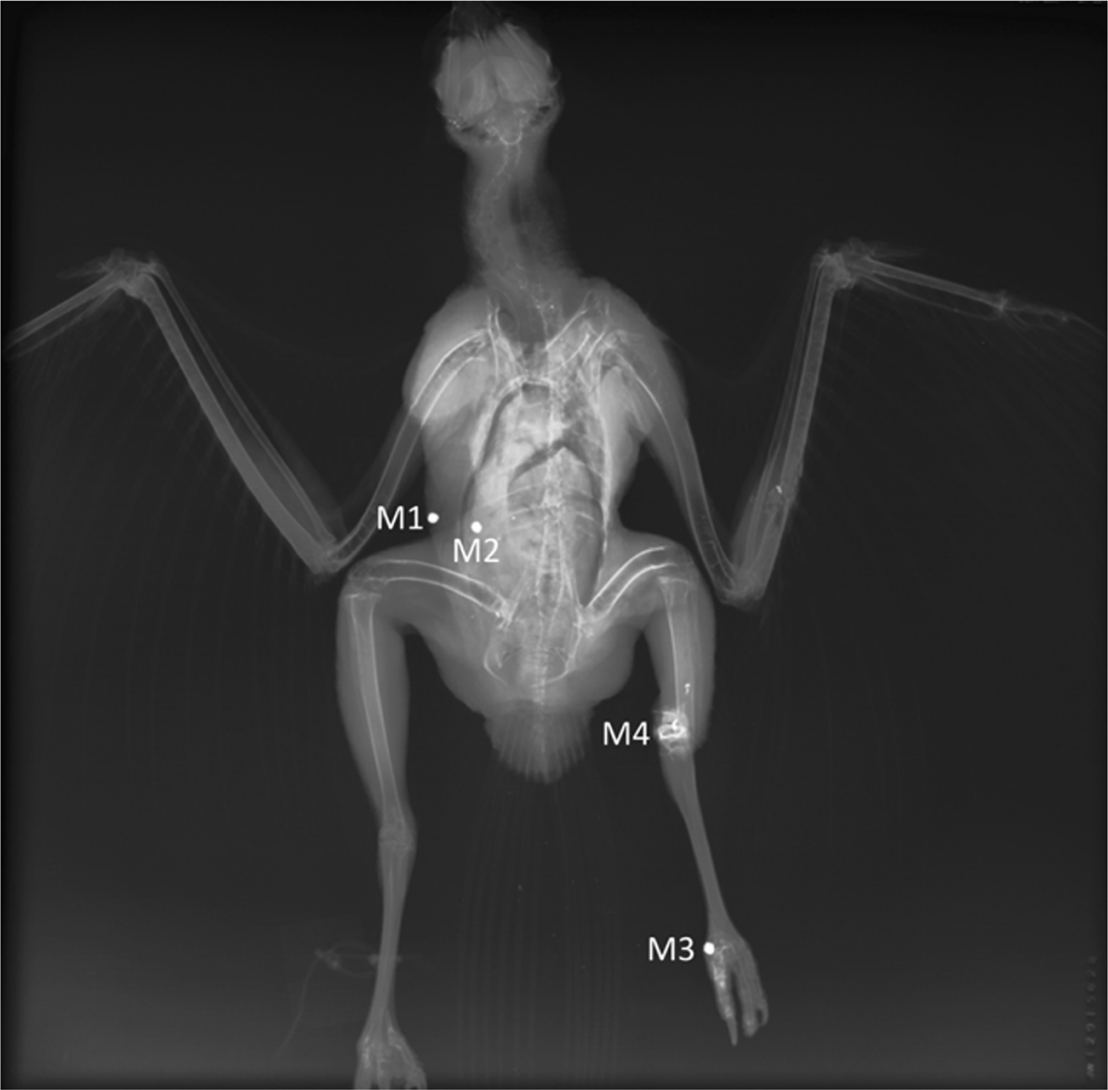
Dorsoventral X-ray image. Metallic foreign bodies in various body parts and multiple bone fractures are visible. Elements M1 – M4 were later isolated from the body for ballistic examination

**Fig. 3:**
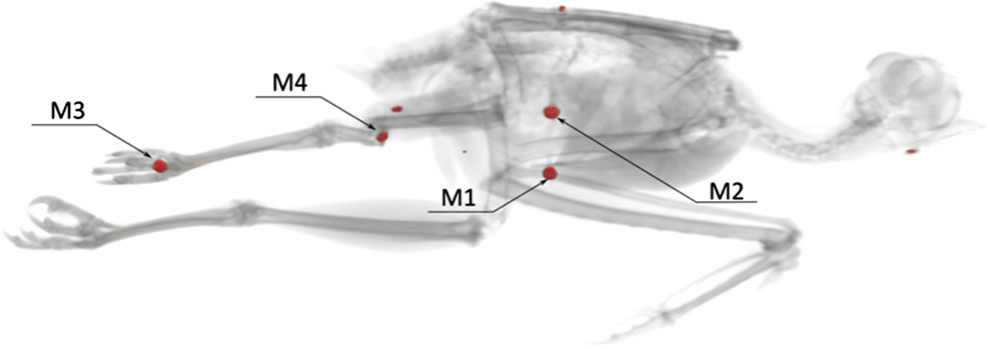
CT reconstruction of a buzzard with metallic foreign bodies colored in red. Elements M1 – M4 were later isolated from the body for ballistic examination

**Fig. 4:**
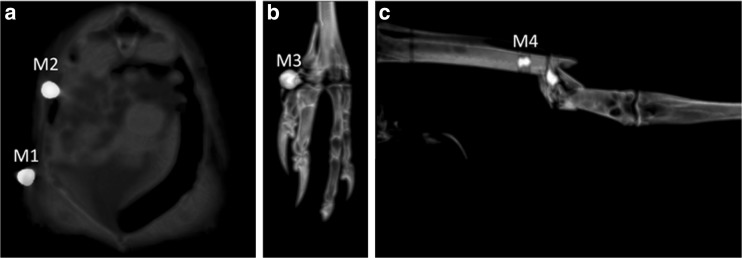
Transverse (**a**), dorsal (**b**) and sagittal (**c**) PMCT images of the thorax (**a**), left metatarsophalangeal joint (**b**) and left tarsus (**c**). Note the foreign bodies M1 – M4 and a fracture of the distal 1/3 of the left tibia (**c**)

X-ray examination revealed multiple bone fractures and metallic foreign bodies in various body parts (Fig. 2). Computed tomographic examination showed numerous, round, sometimes irregular foreign bodies of metal density in different body regions (Fig. 3). In the right zygomatic region there were 7 foreign bodies with a diameter of up to 1 mm. Four foreign bodies, 1-2 mm in diameter, were embedded in the corpora and arches of the 8th and 9th cervical vertebrae and inside the vertebral canal and spinal cord at this level. In 1/3 proximal shaft of the left radius, there were 2 irregular foreign bodies with a diameter of approximately 1 mm and a few even smaller ones. In this area, there was a multiple fracture of the radius and swelling of the surrounding soft tissues. There were several foreign bodies in the thoracic area. The two largest ones had a diameter of 5 mm. One of them was located superficially in the skin (M1), and the other one was in the soft tissues of the chest wall at the level of the right penultimate rib (M2). The remaining numerous elements measured about 1 mm and were located on the right side of the thorax caudally. Two foreign bodies with a diameter of approximately 5 mm were found in the distal 1/3 of the left tibiotarsus (M4), accompanied by a comminuted fracture with displacement and shortening of the bone axis. On the medial surface of the left metatarsophalangeal joint, a foreign body with a diameter of 5 mm was visible (M3). In addition, there were several small 1 mm foreign bodies in the pelvic region and one foreign body in the abdominal area, beyond the outline of the body. Figure 4 shows the location of foreign bodies M1 – M4 in more detail (Fig. 4).

### Ballistic findings

**Fig. 5:**
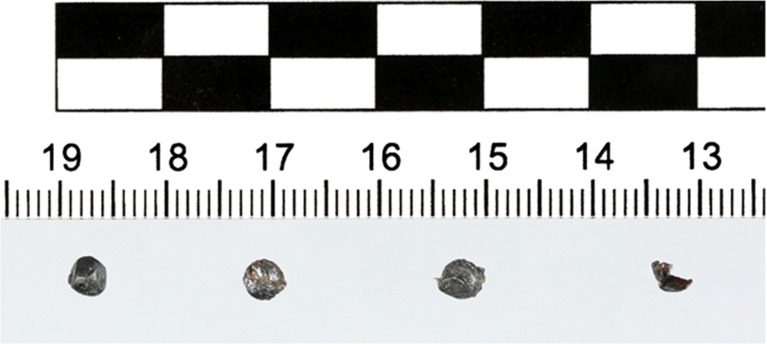
Photograph of elements M1 – M4 (from left to right) isolated from the buzzard

Isolated foreign bodies M1 – M3 were made of a material with an irregular surface of a gray color with a metallic sheen, not showing magnetic characteristics, with low hardness. They had the shape of deformed spheres with a variable diameter of 3.5 – 4 mm and a weight of 0.26 g. Element M4 had the shape of an elongated, arcuate bent strip with a length of about 4 mm, a variable width of about 1 – 2 mm and a thickness of about 0.7 mm (Fig. 5). The features and appearance of elements M1 – M3 indicated that they were lead pellets, constituting a load of shotgun shell, intended for firing from a smoothbore hunting weapon. Their weight indicated that their nominal diameter was 3.5 mm, assuming they were made of a lead alloy with 3% antimony, with a specific gravity of 11.10 g/cm^3^.

## Discussion

**Fig. 6:**
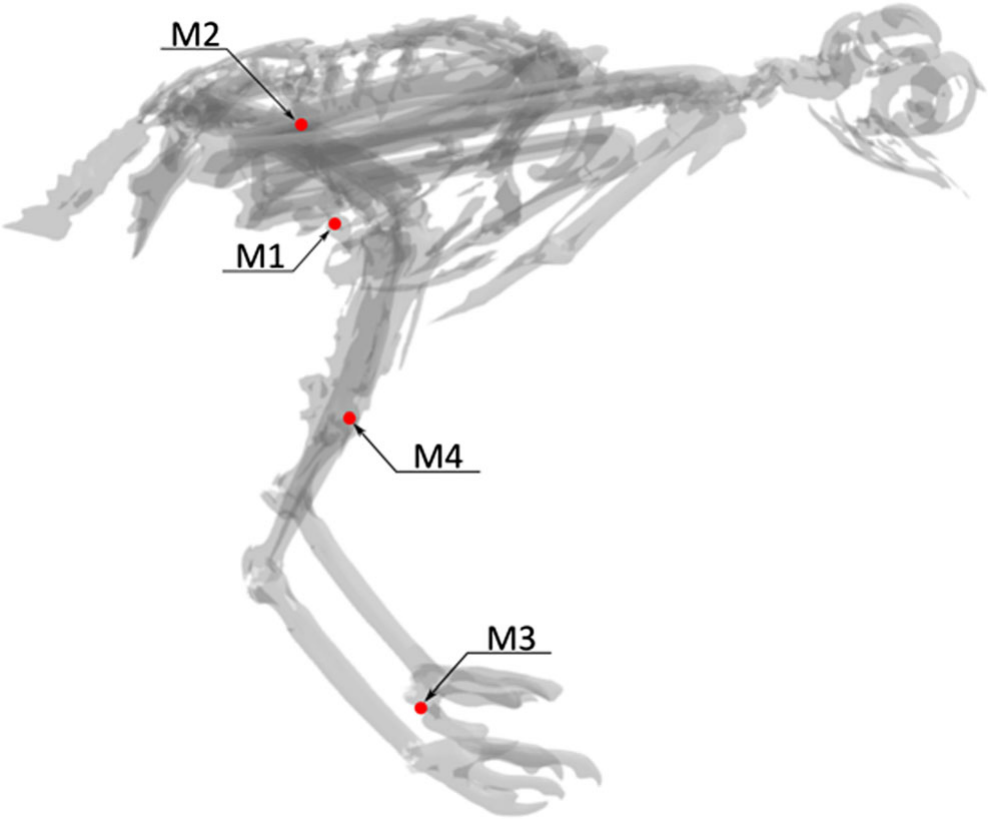
Three-dimensional visualization of the assumed bird position at the time of the gunshot

This buzzard probably died as a result of spinal cord injury at the level of the C8 - C9 vertebrae. The damage was made by a bullet fragment, which presumably originated from disintegration of the M4 bullet on the bones of the knee area. The inlet channels along with the trajectory of M1 and M2 bullets, as well as the location of fragments of the M4 bullet, indicate that the bird had its pelvic limbs bent and the shot came from the right side. This leads to the assumption that the bird was sitting or trying to catch some prey while flying. A sitting position of the bird, however, seems more likely (Fig. 6). The fact that the M3 bullet did not pass through the body and stopped on the proximal phalanx without damaging any bony structure could mean that it lost its energy earlier, perhaps on a tree branch on which the bird was sitting. A small number of pellets isolated from the buzzard and their superficial arrangement indicates that the bird was shot from a considerable distance. A similar diameter of pellets suggests that it was hit from one shot.

Ballistic examination revealed that the shot was taken from a smoothbore hunting gun with a probable 12, 16 or 20 mm caliber, which are the most commonly used calibers in Poland. However, the caliber and the manufacturer of the cartridge from which the isolated pellets have derived could not be unambiguously determined. An assumption can be made that the offender acted deliberately, with the intention of killing the protected bird. The current legal order defined in Polish law provides penalties for such actions. Punishment for breaking this law can be a fine, a restriction of liberty or an imprisonment for up to 2 years. The legislator has imposed a penalty of imprisonment of up to 3 years, if the offense carries the marks of action with particular cruelty [8]. Nonetheless, such cases are very rarely considered by the judiciary in Poland. Collected evidence and lack of knowledge about the circumstances of the incident made it impossible to identify the shooter. In the literature there are only few examples of individual identification in wildlife forensics [9].

Various models of ballistic trauma are available in forensic science, such as ballistic gels, soaps, large animals or tissue analogues [10, 11]. One reason that birds don’t serve as a model is the difference in bone structure to that of humans. The workup presented in this case should be performed shortly after death, since a longer presence of the bullets in the biological material can cause differences of up to 30% in bullet diameter measurements between radiological and ballistic techniques.

In human medicine, post-mortem computed tomography (PMCT) is often used for determining the cause of death and is widely acknowledged as very accurate, especially in cases of traumatic or firearm deaths [12, 13]. PMCT can be successfully used in veterinary medicine as well, and its utilization will grow.

## Key points

1. The cause of death of the buzzard was spinal cord injury at the level of the 8th and 9th cervical vertebrae.
2. A single shot from a smoothbore hunting weapon with a 12, 16 or 20 mm caliber hit the buzzard.
3. There was not enough evidence to identify the perpetrator.
4. PMCT works well in veterinary medicine and should be used more frequently when determining the cause of death of an animal.
